# Chaperone-Based Therapies for Disease Modification in Parkinson's Disease

**DOI:** 10.1155/2017/5015307

**Published:** 2017-08-21

**Authors:** Erik L. Friesen, Mitch L. De Snoo, Luckshi Rajendran, Lorraine V. Kalia, Suneil K. Kalia

**Affiliations:** ^1^Krembil Research Institute, Toronto Western Hospital, University Health Network, 60 Leonard Avenue, Toronto, ON, Canada; ^2^Department of Laboratory Medicine and Pathobiology, University of Toronto, 1 King's College Circle, Toronto, ON, Canada; ^3^Faculty of Medicine, University of British Columbia, 317-2194 Health Sciences Mall, Vancouver, BC, Canada; ^4^Morton and Gloria Shulman Movement Disorders Clinic and The Edmond J. Safra Program in Parkinson's Disease, Division of Neurology, Department of Medicine, Toronto Western Hospital, University Health Network, 399 Bathurst Street, Toronto, ON, Canada; ^5^Division of Neurology, Department of Medicine and Tanz Centre for Research in Neurodegenerative Diseases, University of Toronto, 190 Elizabeth Street, Toronto, ON, Canada; ^6^Division of Neurosurgery, Department of Surgery, University of Toronto, 149 College Street, Toronto, ON, Canada

## Abstract

Parkinson's disease (PD) is the second most common neurodegenerative disorder and is characterized by the presence of pathological intracellular aggregates primarily composed of misfolded *α*-synuclein. This pathology implicates the molecular machinery responsible for maintaining protein homeostasis (proteostasis), including molecular chaperones, in the pathobiology of the disease. There is mounting evidence from preclinical and clinical studies that various molecular chaperones are downregulated, sequestered, depleted, or dysfunctional in PD. Current therapeutic interventions for PD are inadequate as they fail to modify disease progression by ameliorating the underlying pathology. Modulating the activity of molecular chaperones, cochaperones, and their associated pathways offers a new approach for disease modifying intervention. This review will summarize the potential of chaperone-based therapies that aim to enhance the neuroprotective activity of molecular chaperones or utilize small molecule chaperones to promote proteostasis.

## 1. Introduction

Parkinson's (PD) is the second most common neurodegenerative disorder affecting approximately 1% of the population over 60 [1]. People with PD typically present with cardinal motor symptoms including bradykinesia, muscular rigidity, rest tremor, or gait impairment but often also develop nonmotor symptoms, such as cognitive impairment and psychiatric symptoms. Many but not all of the symptoms associated with PD result from loss of the dopaminergic neurons of the substantia nigra pars compacta (SN) [2]. Currently, PD is treated pharmacologically, by enhancing dopamine tone (e.g., dopamine replacement with L-dopa) and, surgically, by deep brain stimulation (DBS) [2]. As the disease progresses L-dopa treatment is associated with disabling complications including motor fluctuation and dyskinesia. DBS is restricted to a select group of patients presenting with L-dopa responsive motor symptoms and L-dopa-induced complications, but without significant cognitive impairment or psychiatric disturbance. Importantly, both interventions only provide symptomatic relief and do not slow the progression of PD. Consequently, there is a need for a treatment addressing the underlying causes of the disease.

Pathologically, PD is characterized by the presence of proteinaceous intracellular aggregates composed primarily of *α*-synuclein, termed Lewy pathology (Lewy bodies and Lewy neurites). Missense mutations and multiplications of the* SNCA* gene, which encodes for *α*-synuclein, cause heritable forms of PD and enhance the propensity of *α*-synuclein to self-aggregate thus implicating *α*-synuclein aggregation in the pathogenesis of the disease [3, 4]. While there is uncertainty regarding the specific form of aggregates (“species”) that are neurotoxic, emerging evidence suggests that *α*-synuclein toxicity is conferred by soluble oligomeric species [5–8]. Given the central role of perturbed *α*-synuclein aggregation in PD, investigation into the nature and modification of the molecular pathways responsible for directing protein folding and misfolding, maintaining proper protein confirmation, and reducing abnormal protein aggregation, presents a promising avenue for identifying a disease modifying strategy.

## 2. Molecular Chaperones

Molecular chaperones are highly conserved proteins that function to maintain proteostasis by directing the folding of nascent polypeptide chains, refolding misfolded proteins, and targeting misfolded proteins for degradation. Molecular chaperones are also termed “heat shock proteins” (HSPs), as initial studies found them to be upregulated in response to high temperatures. In eukaryotes, HSPs are a large and heterogeneous group of proteins that have been classified into families based on their molecular weight: Hsp40, Hsp60, Hsp70, Hsp90, Hsp100, and the small HSPs [20]. The activity of HSP family members is modulated by another class of proteins termed “cochaperones” which can be subdivided based on the presence of a Bcl-2 Associated Athanogene (BAG) domain, a tetratricopeptide (TPR) domain, or a J domain. Each of the families of chaperones and cochaperones are composed of multiple proteins which, despite having similar functions and domain compositions, often vary significantly in terms of their expression pattern and subcellular localization. For a recent review of the complete set of chaperone and cochaperone proteins, see Kampinga and Bergink (2016) [20].

Due to the number and heterogeneity of chaperone and cochaperone proteins, the nomenclature has become complex, with some chaperones receiving multiple names. As such, a new nomenclature was developed where DNAJ, HSPD, HSPA, HSPC, HSPH, and HSPB are the preferred prefix terms for the Hsp40, Hsp60, Hsp70, Hsp90, Hsp100, and small Hsp family members, respectively [21]. For the purposes of this review, “Hsp” will be used when referring to an entire family of Hsp chaperones and the new nomenclature will be used when referring to specific members within a family.

**Figure 1:**
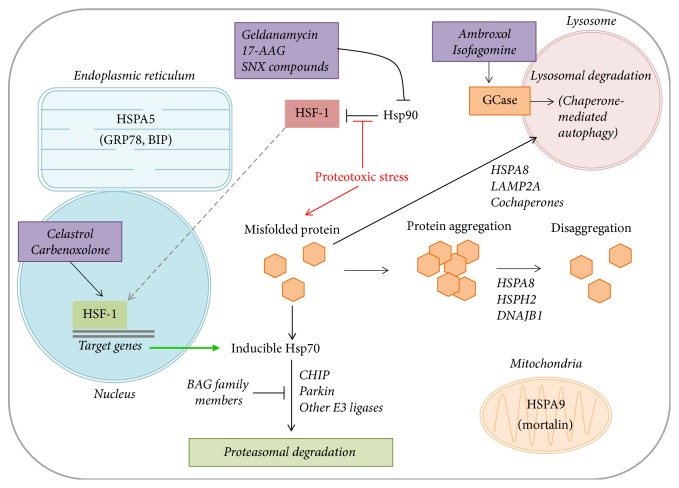
Proposed role of molecular and small molecule chaperones in proteostasis. At baseline, Hsp90 is bound to HSF-1, maintaining its inactive state. In the presence of proteotoxic stress, or the addition of Hsp90 inhibitors (i.e., geldanamycin, 17-AAG, and SNX compounds), active HSF-1 dissociates from Hsp90 and translocates into the nucleus where it induces Hsp70 expression. Inducible Hsp70 family members direct proteasomal degradation through a pathway mediated by CHIP, Parkin, and other E3 ligases. This process is inhibited by BAG family members and promoted by small molecule HSF-1 activators including celastrol and carbenoxolone. In response to proteotoxic stress, chaperones also direct misfolded proteins for degradation via the autophagy-lysosome system, through interactions with various cochaperones (chaperone-mediated autophagy). Chaperone/cochaperone complexes can also function to disaggregate already formed protein aggregates. The pharmacological chaperones, ambroxol, and isofagomine increase glucocerebrosidase (GCase) activity in the lysosome to further promote the process of chaperone-mediated autophagy. Chaperone functions within the endoplasmic reticulum and mitochondria are regulated by the specific members of the Hsp70 family, HSPA5 and HSPA9, respectively.

The two main chaperone machines in eukaryotes are Hsp70 and Hsp90, which together account for at least half of the molecular chaperones present in eukaryotic cells [22]. The Hsp70 family members are the most studied molecular chaperones and have received significant attention in PD due to their abundance in Lewy bodies and their neuroprotective effect in preclinical models of the disease [23]. Only a subset of Hsp70 chaperones, namely, HSPA1A, HSPA1B, and HSPA6, show stress-induced expression patterns, whereas the other Hsp70 family members, such as HSPA8 (often referred to as Hsc70), are expressed constitutively at baseline conditions [20]. A signaling pathway involving the transcriptional activator, heat shock factor 1 (HSF-1), regulates the expression of inducible Hsp70 family members following stressful stimuli (Figure 1). At baseline conditions, HSF-1 is bound by Hsp90, maintaining HSF-1 in an inactive monomeric form [24]. Following proteotoxic stress, HSF-1 dissociates from Hsp90 and translocates to the nucleus where it upregulates transcription of its target genes [25]. Once proteostasis is reestablished, Hsp90 again sequesters HSF-1 into its inactive monomeric form, suppressing inducible Hsp70 expression. This crosstalk between chaperones and the presence of both constitutively active and stress-inducible chaperones on a negative feedback loop allows the cell to execute continuous “house-keeping” tasks in proteostasis, as well as respond to potentially devastating proteotoxic stress.

The primary role of Hsp70 is to ensure proper protein folding. Hsp70 accomplishes this by binding exposed hydrophobic domains on misfolded proteins (“clients”) via its C-terminal substrate binding domain (SBD) and then undergoing multiple ATP hydrolysis cycles at the N-terminal ATPase domain [26, 27]. Hydrolysis of ATP to ADP stabilizes the Hsp70-client complex, which allows Hsp70 to hold the client protein and increases the likelihood of spontaneous refolding [22]. Subsequent ADP-ATP exchange reduces the stability of the Hsp70-client complex, allowing for client dissociation or subsequent ATP hydrolysis cycles. While there are multiple models of the mechanism by which Hsp70 mediates protein refolding, the cycling between ATP and ADP bound states is necessary for this function [28].

The ATP hydrolysis cycle on Hsp70 is modulated by Hsp40, HSPH2 (Hsp110), the TPR domain-containing Hsp70 interacting protein (Hip), and BAG family cochaperone proteins. Hsp40s are important for both client selection and facilitating ATP hydrolysis [29], and Hip stabilizes the ADP bound state of Hsp70 [30]. Both BAG family members and HSPH2 act as nucleotide exchange factors (NEFs), promoting the release of ADP from the ATPase domain [30–32]. As such, both Hsp40 and Hip promote Hsp70-client stability, whereas BAG family proteins and HSPH2 destabilize the interaction. Therefore, the relative abundance of cochaperone proteins may play an important role in the dynamics of Hsp70 refolding activity. A complex interplay between the nature of the client protein, the Hsp70 family member, and the cochaperone proteins present likely determines the efficacy and the mechanism by which a protein becomes refolded.

Outside of their primary function of protein refolding, molecular chaperones also play important roles in cellular processes such as guiding misfolded proteins for degradation through the ubiquitin-proteasome system (UPS) or autophagy-lysosome pathway (ALP), disaggregating protein aggregates, suppressing cell death pathways, and promoting mitochondrial health (Figure 1). Hsp70-mediated protein degradation via the UPS is largely regulated by cochaperone proteins, namely, the C-terminal Hsp70 interacting protein (CHIP), which is both an Hsp70 cochaperone and an E3 ubiquitin ligase, thus providing a mechanistic link between the chaperone system and the UPS [33, 34]. HSPA8 (Hsc70), in conjunction with lysosomal-associated membrane protein 2A (LAMP2A) and multiple cochaperones, can also facilitate protein degradation via the ALP through a process termed chaperone-mediated autophagy (CMA) [35, 36] (Figure 1). Moreover, a chaperone machine composed of Hsp70, HSPH2 (Hsp110), and Hsp40 has a demonstrated “disaggregase” activity by which it can remove misfolded proteins from already formed aggregates [37, 38]. The close relationship between molecular chaperones and protein aggregation has led to their investigation in many neurodegenerative proteinopathies, including PD. 

## 3. Molecular Chaperones in Parkinson's Disease

### 3.1. Molecular Chaperones Modulate *α*-Synuclein Aggregation and Toxicity

Early evidence implicating molecular chaperones in the pathobiology of PD stemmed from the observation by Auluck et al. (2002) that Hsp70 overexpression attenuated *α*-synuclein-mediated dopaminergic neurodegeneration in a* Drosophila* model [39]. This suggests that Hsp70 may play a neuroprotective role in PD. Subsequently, McLean et al. (2002) illustrated that multiple chaperone proteins colocalize with Lewy bodies and that the overexpression of several Hsp40 and Hsp70 family members antagonizes the formation of *α*-synuclein aggregates in vitro [40]. Molecular chaperones were further implicated in the pathobiology of PD by the observation that mutations within the promoter region upstream of both constitutively expressed and inducible Hsp70 family members increase the risk of PD in a patient population [41]. Furthermore, mutations in the mitochondrial Hsp70, HSPA9 (mortalin), were recently suggested to promote the development of PD [42–44]; however, other groups suggest mutations in HSPA9 are not a frequent cause of early-onset PD as they are also found in patient controls [45].

Since these initial studies, the capacity of Hsp70 overexpression to ameliorate *α*-synuclein aggregation and toxicity has been well characterized. Independent groups have shown that Hsp70 overexpression can attenuate *α*-synuclein-mediated cell death in yeast [46] and reduce high molecular weight aggregates and toxicity in rodent models of PD [47, 48]. Hsp70 overexpression was shown to be protective against cell death mediated by the mitochondrial complex I inhibitor, 1-methyl-4-phenyl-1,2,3,6-tetrahydropyridine (MPTP), both in vitro [49] and in vivo [50]. Although *α*-synuclein aggregation is not a feature of this toxin model, *α*-synuclein is required for MPTP-induced cell death as demonstrated by the resistance of *α*-synuclein null mice to MPTP [51]. Mitochondrial HSPA9, however, may play a role in the mitochondrial defects caused by the pathological A53T mutant *α*-synuclein as HSPA9 knockdown protects against the mitochondrial fragmentation and increased susceptibility to the complex I inhibitor, rotenone, induced by A53T overexpression [52].

In parallel with the Hsp70 overexpression results, recent studies have demonstrated that microRNA (miRNA) mediated translational repression of Hsp70 exacerbates *α*-synuclein aggregation and toxicity in vitro [53] and that miRNAs targeting Hsp70 are upregulated in brain regions with Lewy pathology [54]. Furthermore, the Hsp70 family members HSPA8 (Hsc70) and HSPA9 have lower expression in the SN (HSPA8/9) [55] and leukocytes (HSPA8) [56, 57] of PD patients relative to healthy controls, suggesting that chaperone levels and function may have a role in the pathogenesis of PD.

In contrast, the endoplasmic reticular Hsp70 family member, HSPA5 (GRP78/BiP), was found to be more abundant in the cingulate gyrus and parietal cortex of individuals with Dementia with Lewy Bodies (DLB) or PD with Dementia (PDD) relative to individuals with Alzheimer's disease (AD) and healthy controls [58]. The increase in HSPA5 in the cingulate gyrus was positively correlated with *α*-synuclein abundance, leading the authors to suggest that HSPA5 may be upregulated to mitigate *α*-synuclein toxicity [58]. This notion is supported by the observations that miRNA-mediated HSPA5 depletion enhances rotenone-induced cell death in vitro [59], and HSPA5 knockdown exacerbates the toxicity of AAV-delivered *α*-synuclein in rats [60]. Moreover, multiple studies have demonstrated that HSPA5 overexpression can suppress *α*-synuclein aggregation and toxicity in vitro and in vivo [61, 62].

The mechanism by which Hsp70 attenuates *α*-synuclein aggregation and toxicity seems to be dependent on both its refolding activity and its function in protein degradation via the UPS and ALP. Mutations that alter the ATPase function of Hsp70 (K71S) abolish its protective effect on *α*-synuclein toxicity, indicating that Hsp70 folding activity is necessary for its protective function [48]. Interestingly, this mutation has no effect on the capacity of Hsp70 to suppress *α*-synuclein aggregation [48], suggesting that Hsp70 uses distinct mechanisms to attenuate either the aggregation or the toxicity of *α*-synuclein. In addition to antagonizing the aggregation of *α*-synuclein, Hsp70 may also facilitate the disaggregation of already formed *α*-synuclein aggregates, similar to the Hsp70 “disaggregase” activity that has already been well characterized in other models of protein aggregation [38]. For example, Gao et al. (2015) recently demonstrated that an Hsp70 machine composed of HSPA8, DNAJB1, and HSPH2 could effectively disassemble preformed *α*-synuclein fibrils in vitro and in* C. elegans* [37] (Figure 1).

Hsp70/cochaperone complexes also mitigate *α*-synuclein-mediated toxicity by promoting the degradation of misfolded *α*-synuclein via either the UPS or ALP. Several studies have suggested that CMA may be playing an important role in mitigating *α*-synuclein toxicity and aggregation [35, 63, 64]. Enhanced *α*-synuclein expression in both transgenic and paraquat models of PD results in a concurrent enhancement of LAMP2A and HSPA8 expression and a greater movement of *α*-synuclein into the lysosomes [63]. Moreover, both LAMP2A and HSPA8 have lower expression in the SN of PD patients [55], and a recent study demonstrated a correlation between the loss of LAMP2A and *α*-synuclein aggregation in postmortem PD brains [65]. Interestingly, the observed decrease in LAMP2A and HSPA8 expression anatomically overlaps with an increase in miRNAs capable of translationally repressing both LAMP2A and HSPA8 [54], further implicating miRNAs in PD-associated chaperone dysregulation.

Outside of CMA, the Hsp70 cochaperone, CHIP, plays an important dual function in *α*-synuclein degradation, as it can target *α*-synuclein for degradation by either the proteasome or lysosome via its TPR domain or U-box domain, respectively [66]. CHIP may mediate this through ubiquitination of *α*-synuclein and suppression of oligomer formation [67]. However, not all Hsp70 cochaperones promote *α*-synuclein degradation. In contrast, overexpression of the BAG family member, BAG5, antagonizes CHIP-mediated *α*-synuclein ubiquitination, which prevents the ability of CHIP to suppress oligomer formation [67] and also enhances *α*-synuclein-mediated toxicity [68]. Therefore, the balance between multiple cochaperones may assist Hsp70 in triaging whether to refold or degrade a client substrate, and a disruption in the relative abundance or activity of cochaperones may compromise the chaperone system and subsequently proteostasis.

Taken together, the capacity of Hsp70 and its cochaperones to refold, disaggregate, and target for degradation potentially toxic *α*-synuclein species suggests that molecular chaperones may have a central and multifaceted role in the pathobiology of PD. Since multiple chaperones are downregulated, sequestered into protein aggregates, or face age-related loss-of-function in the brains of people with PD, it is possible that the depletion and dysfunction of molecular chaperones may further contribute to the progression of PD.

### 3.2. Molecular Chaperones and Other PD-Relevant Proteins

The potential role of chaperones in the pathobiology of PD is broadened by their capacity to regulate the stability and function of PD-relevant proteins other than *α*-synuclein, including LRRK2* (PARK8)*, PINK1* (PARK6)*, parkin* (PARK2)*, and DJ-1* (PARK7)*. LRRK2 plays a regulatory role in vesicular trafficking, microtubule dynamics and mitochondrial health [69]. Mutations in LRRK2 are associated with autosomal dominant PD, and common genetic variants are associated with an increased risk of developing sporadic PD [70]. Pathological mutations in LRRK2 are associated with autophagy dysfunction (including CMA dysfunction), proteasome dysfunction, and mitochondrial stress. The pathogenic G2019S, R1441C, and Y1699C LRRK2 mutations were shown to enhance the clearance of the trans-Golgi network (TGN) via a protein complex including the chaperone proteins Hsp70 and BAG5 plus Rab7L1 and Cyclin G Associated Kinase (GAK), which are both located in risk loci for sporadic PD [71]. TGN dynamics have a close relationship with the ALP suggesting that this chaperone-dependent clearance of the TGN by LRRK2 could explain how pathogenic LRRK2 mutations disrupt autophagy. CHIP and Hsp90 have been shown to play important and opposing roles in regulating LRRK2 stability, as CHIP mediates the ubiquitination and proteasomal degradation of LRRK2, whereas Hsp90 stabilizes it [72]. Ko et al. (2009) demonstrated that the toxicity of mutant LRRK2 could be enhanced by CHIP knockdown and attenuated by CHIP overexpression. Moreover, Hsp90 inhibition with the pharmacological agent 17-AAG (discussed below) was also protective against mutant LRRK2-mediated toxicity [72], presumably by promoting the degradation of the toxic gain-of-function mutant proteins. The G2385R LRRK2 variant is a risk factor for PD. G2385R LRRK2 demonstrates increased binding to Hsp90 and enhanced CHIP-dependent degradation resulting in lower steady state levels compared to wild-type LRRK2 [73]. Taken together, these results suggest that the interaction between chaperones and LRRK2 may regulate LRRK2 function, and these interactions may be compromised with PD-related mutations or variants of LRRK2.

Hsp70 and Hsp90 family members also regulate the stability of PINK1 and Parkin. PINK1 and Parkin function together in a pathway responsible for the selective autophagic clearance of damaged mitochondria, a process termed mitophagy [74]. The E3 ubiquitin ligase activity of Parkin also facilitates proteostasis via the UPS. Hsp90 regulates the processing and stability of PINK1, and the Hsp90 family member HSPC5, commonly known as TNF Receptor Associated Protein 1 (TRAP1), promotes mitochondrial health and compensates for the mitochondrial dysfunction caused by PD-associated PINK1 mutations [75]. Conversely, PINK1 and parkin mediated mitophagy protects cells against increased susceptibility to mitochondrial stress that results from the knockdown of mitochondrial HSPA9 [76, 77]. HSPA1L and the cochaperones, BAG2 and BAG4, have all been shown to modulate PINK1-Parkin mediated mitophagy [78, 79]. Outside of mitophagy, Hsp70 supports Parkin by preventing it from being sequestered [68] and acts in concert with CHIP to promote the E3 ubiquitin ligase activity of Parkin following proteotoxic stress [80]. In contrast, the cochaperone BAG5 inhibits Parkin E3 activity, which may provide a mechanistic explanation as to how BAG5 enhances dopaminergic neurodegeneration [68].

Molecular chaperones have also been shown to interact with DJ-1. Upregulation of DJ-1 results in a concurrent increase in Hsp70 expression [81], and PD-associated DJ-1 mutations enhance the association of DJ-1 with cytosolic Hsp70, HSPA9, and CHIP [82]. Furthermore, a recent study demonstrated that the cochaperone BAG5 interacts with DJ-1 and decreases its stability [83]. In turn, BAG5 suppresses the protective effect of DJ-1 on cell death caused by rotenone [83].

In summary, chaperones not only modulate *α*-synuclein but are implicated in multiple pathways that mediate the pathobiology of PD. Significant progress has been made in terms of understanding how chaperones and cochaperones can be manipulated to attenuate or reverse PD pathology. More recently, a mutation in J domain-containing cochaperone, DNAJC13, has been identified as a cause of autosomal dominant PD, further supporting a potentially important role for chaperone proteins in the pathogenesis of PD [84]. Considering their ability to protect against *α*-synuclein aggregation and neurodegeneration in preclinical models, as well as their effects on other PD-related proteins, the chaperone systems represent a suitable target for the design of novel therapeutics that have the potential to slow the progression of PD.

## 4. Potential Chaperone-Based Strategies for Treatment of PD

### 4.1. Small Molecule Chaperones

Small molecule chaperones are low molecular weight compounds that exhibit their own chaperone function by enhancing protein stabilization and folding processes and by antagonizing protein aggregation [10, 85]. These compounds are distinct from molecular chaperones in that they are neither proteins nor components of the cell's primary response mechanism to proteotoxic stress. Small molecule chaperones are subdivided into two groups: chemical chaperones and pharmacological chaperones [10]. Chemical chaperones are classified as either osmolytes or hydrophobic compounds and typically promote protein folding nonspecifically by creating a chemical environment that encourages proteins to acquire the proper conformation [10]. In contrast, pharmacological chaperones bind directly to their target protein(s) to modulate its conformation and stability [10, 85].

Osmolyte chemical chaperones include free amino acids and their derivatives, polyols, and methylamines. They are often enriched in conditions of environmental stress and denaturation to promote protein homeostasis and quality control processes [86]. Examples of relevant osmolytes include trehalose and mannitol. Oral 2% trehalose solution has demonstrated high effectiveness in a mouse model of Huntington's disease (HD) [87]. Similar to PD, HD is a neurodegenerative movement disorder associated with protein aggregation. Specifically, trehalose treatment resulted in decreased aggregation of the protein implicated in HD, huntingtin, and improved motor dysfunction [87]. More recently, it was shown that 2 and 5% oral trehalose solutions ameliorate the behavioural deficits and neurochemical pathology associated with a preclinical rat *α*-synuclein PD model [88]. Mannitol, which is currently widely used clinically as an FDA-approved osmotic diuretic [16] (Table 1), can reduce *α*-synuclein aggregation in vitro, in* Drosophila*, as well as in the hippocampus, basal ganglia, and SN of transgenic mouse models of PD [17, 89]. Moreover, mannitol-mediated reduction of *α*-synuclein aggregation correlates with significant neuroprotection and correction of behavioural deficits [17, 89]. The hydrophobic compound 4-phenylbutyrate (PBA) is another FDA-approved drug that serves as a chemical chaperone with beneficial in vitro and in vivo effects on *α*-synuclein aggregation and neurodegeneration [90]. This compound can be given via oral supplementation and is currently used for urea cycle disorders [17]. Though PBA can penetrate the blood brain barrier (BBB), work with HD mouse models has demonstrated that high doses are required to achieve benefits, which would likely translate to the maximum tolerability dosage for humans [18].

Pharmacological chaperones, such as ambroxol and isofagomine, can cross the BBB and have been demonstrated to increase the enzymatic activity of glucocerebrosidase (GCase) [91] (Figure 1). Mutations in the* GBA* gene, which encodes for GCase, are associated with an elevated risk of developing PD and decreased GCase activity in lysosomes. This reduction in GCase activity is associated with increased *α*-synuclein aggregation likely due to impairment of the ALP [92]. By enhancing GCase activity, pharmacological chaperones reduce *α*-synuclein accumulation in vitro and in the SN of mice [91, 93, 94]. Like chemical chaperones, pharmacological chaperones also require high doses to be beneficial which may limit their treatment efficacy.

### 4.2. HSF-1 Modulators

Endogenous molecular chaperone function can be modulated pharmacologically with compounds that augment endogenous chaperone levels. Several HSF-1 modulators including celastrol and carbenoxolone can trigger HSF-1 activation, leading to downstream induction of Hsp70 expression [9] (Figure 1). Celastrol has been demonstrated to be effective against protein aggregation and toxicity in various neurodegenerative disease models, including dopaminergic neuroprotection in a* Drosophila* model of PD [95]. However, this compound has been tested in short-term clinical trials for rheumatoid arthritis [10], and its clinical applicability may be restricted due to its toxicity [9]. Carbenoxolone has demonstrated the ability to attenuate *α*-synuclein and ubiquitin aggregation in vitro and in vivo [13, 96, 97]. Thus, it may have potential as a chaperone-mediated therapeutic option for PD. Carbenoxolone has reached phase II clinical trials in the UK for psoriasis treatment [10] so some safety and tolerability data should soon be available.

### 4.3. Hsp90 Inhibitors

The naturally occurring small molecule antibiotic, geldanamycin (GA), inhibits the interaction between Hsp90 and HSF-1, leading to increased Hsp70 expression [11] (Figure 1). In vitro cell studies have demonstrated the capability of this compound to decrease *α*-synuclein aggregation and reduce cell toxicity [98], and its neuroprotective effects have been shown in* Drosophila* and MPTP mouse models of PD [14, 99]. However, translation of this drug to the clinical setting is prevented by its in vivo toxicity, poor solubility, and limited penetration through the BBB [13, 14]. Other analogues of GA include 17-AAG and 17-DMAG, which similarly prevent *α*-synuclein aggregation and toxicity, but are more potent and less toxic than GA [12, 100]. However, 17-AAG and 17-DMAG were both tested in separate clinical trials relating to cancer treatment and were discontinued due to hepatotoxicity and limited efficacy [101]. Moreover, 17-AAG has poor permeability of the BBB, limiting its pharmacological usage for neurodegenerative diseases [13, 15] (Table 1). Consequently, compound library screening for small molecule Hsp90 inhibitors with improved pharmacokinetics, including BBB permeability, have led to the identification of SNX compounds [13]. These compounds are associated with an increase in Hsp70 activity in the brain and a reduction in *α*-synuclein oligomerization and toxicity in vitro [12]. An in vivo study using a rat model of PD has also demonstrated benefits of these compounds on rescuing striatal dopamine levels but not dopaminergic cell loss [102]. Although preclinical work suggests that there is therapeutic potential for the use of these compounds in PD, further drug development is required before translation to clinical trials.

### 4.4. Gene Therapy

Gene therapy represents a nonpharmacological approach to enhance chaperone function by exogenously elevating chaperone levels. Viral vectors (including adeno-associated virus (AAV) and lentivirus) have been demonstrated to be more efficient than nonviral vectors for gene delivery [103] and have been widely used to efficiently transduce postmitotic cells such as neurons, providing stable long-lasting expression [104]. AAV vectors are nonreplicating, rarely integrate, elicit minimal inflammation or toxicity in the brain, and do not induce disease, making it safe for clinical use [105–107]. Furthermore, intrinsic properties of the vector as well as the use of specific promoters can be engineered to regulate gene expression levels and cell-specificity [108].

Viral-mediated overexpression of chaperones has been demonstrated to increase survival of dopaminergic neurons in preclinical rodent models of PD [36, 39, 47, 50, 68, 109]. Another chaperone molecule with potential for gene therapy is the yeast, Hsp104, which has demonstrated disaggregase capacity [110]. Jackrel et al. (2014) engineered a highly active Hsp104 mutant that disassembles preformed protein aggregates from preexisting inclusions more rapidly and suppresses dopaminergic neurodegeneration in* C. elegans* more effectively than native Hsp104 [111]. Moreover, lentiviral delivery of yeast Hsp104 to the SN in a rat model attenuated *α*-synuclein toxicity [110], suggesting a similar approach could be taken in human patients. It should be noted, however, that although AAV vectors themselves elicit minimal immune response, foreign transgenic proteins may result in astrocyte and microglia activation with neuroinflammation and a potential neurotoxic response [112]. This can potentially limit the delivery of more specific or efficacious reengineered proteins, such as Hsp104.

Several clinical trials have demonstrated the safety of AAV- and lentivirus-mediated gene delivery in humans with PD [19] (Table 1). Although these trials mostly overexpress neurotrophic factors or deliver enzymes to enhance dopamine production, they provide proof-of-principle that chaperones could be modulated using viral vectors in humans. An alternative, less invasive approach for gene delivery involves the use of magnetic resonance imaging-guided focused ultrasound (MRIgFUS) to open the BBB. This method can be combined with the IV administration of a liposome-microbubble conjugated system containing genetic material, which allows for the targeted transfection of specific neuroanatomical regions [113]. MRIgFUS has been used in rodent models for gene delivery to the SN [113, 114]. Since the pathology of PD is not limited to the SN, viral delivery to multiple brain regions may be required for effective chaperone-based therapies. The minimally invasive nature of MRIgFUS may make it a more feasible delivery strategy than stereotactic injections.

## 5. Conclusions

Given the significant amount of evidence implicating molecular chaperones in the pathobiology of PD, this family of proteins may be a rational target in the design of novel therapeutics. While there is a high degree of complexity in molecular mechanisms of the Hsp70 and Hsp90 chaperone machines and the cochaperone proteins that regulate them, preclinical studies have clearly demonstrated that these proteins can be specifically and effectively targeted to slow or prevent disease progression. Currently, the major obstacle in applying these therapies to the patient population has been toxicity and reduced BBB penetrance. As such, gene therapy has emerged as a viable method by which to modulate chaperone activity within the brain. Preclinical and clinical trials have demonstrated the efficacy of intracranial gene delivery using viral vectors, indicating that this is a safe and effective method to specifically target molecular chaperones. Novel minimally invasive techniques, such as BBB permeabilization using MRIgFUS, represent a means by which pharmacological and genetic chaperone therapy delivery can be optimized, while minimizing the risk conferred to the patient. Significant work remains to be done in the preclinical domain to optimize methods to target chaperone proteins but the potential for the development of a novel therapeutic approach that slows neurodegeneration in PD remains high.

## Figures and Tables

**Table 1:** Examples of relevant therapeutics that either target endogenous molecular chaperones, exert their own chaperone function, or have promise for applying chaperone therapies in humans and their progress in preclinical research and clinical trials (CTs).

Chaperone therapies	Compounds	Current clinical trials (CTs)	Clinical utility
HSF-1 modulators			

Trigger HSF-1 activation induces downstream Hsp70 expression [9]	Celastrol	Short-term CTs for rheumatoid arthritis [10]	Limited: strong human toxicity [9]
	Carbenoxolone	Phase II CTs in UK for psoriasis [10]	Potential: trials in PD patients needed

Hsp90 inhibitors			

Inhibits the interaction between Hsp90 and HSF-1, leading to increased Hsp70 expression and activity [11, 12]	Geldanamycin		Limited: in vivo toxicity, poor solubility, and BBB penetration [13, 14]
	17-AAG	CTs for cancer treatment, discontinued	Limited: poor BBB penetration [13]
	17-DMAG	CTs for cancer treatment, discontinued	Limited: human toxicity [15]
	SNX-2112		Potential: trials in PD patients needed

Chemical chaperones			

Nonspecific compounds that benefit protein stabilization and folding and antagonize protein aggregation [10]	Osmolytes (i.e., 2% trehalose, mannitol)	Mannitol is FDA-approved osmotic diuretic [16]	Limited: high concentration dose likely needed for use in PD patients
	Hydrophobic compounds (i.e., 4-PBA)	4-Phenylbutyrate is FDA-approved, currently used for urea cycle disorders [17]	Limited: HD mouse model indicates needing high doses near max tolerability for human benefits [18]

Pharmacological Chaperones			

Specifically bind target protein for chaperone-mediated proteostasis [10]	Pharmacological chaperones (i.e., ambroxol, isofagomine)		Limited: high doses likely required for benefits in PD patients

Gene therapy			

Nonpharmacological modulation of chaperones	Adeno-associated virus vector of gene delivery	Several CTs for viral-mediated gene delivery in PD patients	Potential: safety of gene therapy has been established in PD patients [19]. It will require identification of appropriate chaperone targets
